# Modeling and predicting water consumption in fattening pigs using autoregressive moving average with external parameters

**DOI:** 10.1038/s41598-026-40343-7

**Published:** 2026-02-20

**Authors:** Maria Marroco, Amanda Fernández-Fontelo, Joaquim Segalés, Beatriz García-Morante

**Affiliations:** 1 IRTA, Animal Health, Centre de Recerca en Sanitat Animal (CReSA), Universitat Autònoma de Barcelona (UAB), Campus, Bellaterra, 08193 Catalonia Spain; 2 Unitat mixta d’investigació IRTA-UAB en Sanitat Animal, Centre de Recerca en Sanitat Animal (CReSA), Campus de la Universitat Autònoma de Barcelona (UAB), Bellaterra, 08193 Catalonia Spain; 3 Departament de Matemàtiques, Universitat Autònoma de Barcelona (UAB), Bellaterra, 08193 Spain; 4 Departament de Sanitat i Anatomia Animals, Facultat de Veterinària, Universitat Autònoma de Barcelona (UAB), Campus de la UAB, Bellaterra, 08193 Spain; 5 World Organization for Animal Health (WOAH) Collaborating Centre for the Research and Control of Emerging and Re-Emerging Swine Diseases in Europe (IRTA-CReSA), Barcelona, Spain

**Keywords:** Water, Drinking pattern, Pig, Model, ARMA, Time series, Scientific data, Animal behaviour, Computer modelling, Time series

## Abstract

The effective monitoring of water consumption patterns is crucial to enhance health and welfare in pig production. Understanding its dynamics and influencing factors may support early detection of welfare issues and optimize farm management. This study analysed historical water consumption data in fattening pigs and developed a predictive model to capture these dynamics. A retrospective study was conducted using thirty time series of hourly water consumption records collected over a three-year period from five batches of fattening pigs. An autoregressive moving average model with exogenous variables (ARMAX) was used to account for the autocorrelated structure of the data and to include environmental and physiological covariates such as temperature, ammonia concentration and fattening day. Water usage progressively increased throughout the fattening period, following a circadian rhythm. External factors, particularly ambient temperature, significantly affected both long-term trends and daily fluctuations. Two distinct consumption patterns were observed depending on farm temperature conditions. The ARMAX model demonstrated strong accuracy in predicting water usage, effectively capturing trends and deviations in water use. Water consumption in fattening pigs is shaped by internal time patterns and environmental factors. Using an ARMAX model, we integrated autoregressive and moving average components with key covariates to accurately predict hourly usage. This approach demonstrates the value of combining diverse data streams to enhance animal health and welfare management.

**Supplementary Information:**

The online version contains supplementary material available at 10.1038/s41598-026-40343-7.

## Introduction

Pork meat is in high demand globally and is projected to increase in the coming years^1,2^. At the same time, rising concerns about animal well-being, food safety, and public health represent growing challenges for the pig sector, especially in the pursuit of sustainable breeding systems. Precision livestock farming (PLF) offers significant opportunities through real-time monitoring and management technologies aimed at improving animal care and reducing the environmental footprint of livestock production^3^. For instance, sensor-based systems can play a key role by detecting animals in need of attention and generating timely alerts for farm staff, allowing for prompt interventions to address potential issues before they escalate. Such early warning systems have been developed over the past two decades for various livestock conditions, such as detecting heat stress in cattle^4^, clinical mastitis in cows^5^or tail biting in pigs^6^. Another area of increasing interest is the use of sensors to model changes in animal behaviour, as such changes may precede or accompany clinical signs of disease or injury and affect animal performance^7–9^.

On this regard, the importance of monitoring water consumption in pigs has become evident during the last decade^7^. In a recent study exploring veterinarians’ views on data utilisation for pig health and welfare management, the potential use of water consumption data as health indicator to generate alerts was emphasised by participant stakeholders^10^. Normally, pigs exhibit a stable daily water consumption pattern, but deviations may indicate disease^11,12^, feed issues, water loss and/or ventilation problems^13^. Thus, a reliable real-time water consumption monitoring system may be an asset in pig rearing^7^. Despite its significance, few studies modelling pig water consumption patterns have been published^14^. A common limitation across studies is the lack of dynamic analysis, as water intake predictions are calculated through simple equations^15,16^., making it difficult to detect changes in behaviour over time.

According to Madsen & Kristensen^11^, water consumption must be measured in discrete intervals and hourly sums are preferable for monitoring purposes. Predicting water consumption on an hourly basis requires models that can capture short-term trends, daily recurring patterns specific to animal behaviour periodicities as well as temporal correlation structures related to the inherent nature of the process being modelled. While a dynamic linear model (DLM) has been previously proposed for modelling the drinking patterns of young pigs^14^, to the authors knowledge, an Auto-Regressive Moving Average (ARMA) model could be well-suited for this task too. ARMA models are part of the classical time series models, and are widely applied in animal epidemiology for describing mortality patterns and conducting disease surveillance^17–20^, as well as for detecting changes in overall trends, including disease outbreak detection^21,22^or antimicrobial resistance predictions^23^.

For water consumption to serve as a criterion to differentiate disease from other influencing factors, a more comprehensive investigation of water usage patterns that includes additional variables apart from water consumption is needed. For instance, environmental factors, such as temperature, or feeding schedules have previously been reported to influence pig’s drinking behaviour^16,24^. The effect exerted by the abovementioned variables over water consumption could be properly considered using a natural extension of the ARMA model (ARMAX), which allows for the inclusion of covariates to model a non-stationary time series process. On top, if trends and seasonal patterns are observed, e.g., hourly fluctuations, covariates related to these can also be included in an ARMAX-type process^25^.

Altogether emphasizes the value of adding knowledge background about water consumption behaviour in pigs to better interpret eventual disturbances in common patterns and, thus, act as a potential warning of on-farm undesired events. In this context, the aim of the current work was twofold. First, based on an ARMAX model structure, to describe the water consumption pattern of fattening pigs, including the average daily pattern, patterns related to trends and seasonal components, as well as potential structures of autocorrelation of the water consumption process of fattening pigs. Second, to use the estimated ARMAX model as a tool for short-term prediction of the expected hourly water consumption.

## Materials and methods

### Farm, animals and data collection

Data was obtained from a fattening farm located in the province of Lleida (Northeastern Spain). It consisted of three separated buildings, with two barns (units) each connected by a central corridor. All units were managed all-in/all-out. Each barn had 56 pens of 9 m^2^ housing 12–13 fattening pigs. Pens had fully slatted concrete floors; floor space was 0.7 m^2^/pig approximately and there was one feeder and one drinker per pen. Pigs were fed a standard cereal-soy diet *ad libitum*. The farm complied with animal welfare regulations under Spanish legislation Royal Decree 159/2023 (Real Decreto 159/2023, de 7 de marzo).

All measurements were obtained through fattening periods (batches) of 15 weeks, with pigs with an average starting body weight of 23.5 kg up to 110 kg of body weight at slaughter time. Water consumption data and environmental records were collected hourly at barn level from several batches. Environmental variables included temperature (°C) and relative humidity (%), ammonia (NH₃, ppm), and carbon dioxide (CO₂, ppm). All environmental measurements were obtained using commercial sensors from the same manufacturer (Exafan, Spain). Temperature was measured using a temperature probe with an accuracy of ± 0.5 °C, ammonia concentrations were monitored using DOL 53 sensors with an accuracy of 1.5 ppm or ± 10% of the measured value, and carbon dioxide concentrations were recorded using DOL 119 sensors. All environmental sensors were installed in the barn corridors to capture the general indoor conditions. A laser-based sensor was used to monitor feed levels in the farm silos. While daily measurements were relatively consistent for feed consumption, the hourly data exhibited high variability and lacked the precision required for the intended temporal resolution of the model. Consequently, this variable—despite its potential relevance—was excluded from the analysis.

Specifically, the study period comprised data from five consecutive batches (from December 2020 to January 2023) for each of the six barns. Further details on each batch are provided in Table S1 in the Supplementary Materials. Henceforth, a total of 30 time-series of water consumption were obtained. Additionally, our database contained the corresponding values of environmental variables mentioned above for each time step. Considering that each barn and batch housed approximately 690 pigs, the dataset comprised 77.538 barn-level observations, from which average values per pig were derived from aggregated barn-level measurements.

### Farm’s drinking water distribution system

Growing pigs were enabled to drink *ad libitum*. Water was provided by stainless steel pig automatic drinking bowls with nipples in each pen and the water flow rate was adjusted to 1 L/min. On the farm, a total of 123 automated digital water flowmeters (iPerl 50 R800, Sensus) were distributed across different areas. Some flowmeters monitored the water treatment process, including general consumption and treated water, the latter only for farm management purposes, while others tracked water usage in specific areas such as cleaning operations, cooling systems, and each barn individually. Of these, 112 flowmeters were dedicated exclusively to measuring the water consumed in the barns. Most flowmeters were located in mixed or shared zones of each barn and in the water treatment area, whereas the flowmeters assigned to individual barns were installed directly to each barn. The computerized system responsible for collecting consumption data received a summary of the flowmeter readings approximately every 20 min by calculating the difference in the amount of water registered between two consecutive readings. Once this data was incorporated into the database, it was grouped to provide an hourly summary of recorded consumption for the same day.

### Data transformation and analysis

Concerning on-farm water use, most of the water consumption was for drinking, representing 92.6% of total observations. Importantly, water usage data for cooling and cleaning purposes were uniquely available in one of the farm buildings, accounting for 7.4% of total observations. In any case, cooling and cleaning water data were removed from the dataset and therefore not used in the model construction. In addition, those time points with missing values, where water usage values and the number of pigs could not be paired, were also discarded. Lastly, drinking data contained outliers (approximately 7.48% of the observations per barn) due to random sensor reading errors across the time series (e.g., negative values or sums of readings of consecutive day hours), which were removed from the database. Specifically, values exceeding 10 L/pig/hour were excluded, as such levels fell well beyond the upper tail of the empirical distribution (above the 99th percentile), indicating extreme and likely erroneous readings. Further details regarding the cleaning process of the database are provided in Table S2 in Supplementary Materials.

The number of pigs per batch was not constant over time because each batch initially consisted of a different number of pigs (ranging from 660 to 730) and this number was affected by the mortality rate, which averaged 3.39% ± 1.58% in the study period. Consequently, the total water consumption data of the barn was divided by the number of pigs in the respective barn at a respective time point, so that the final units of the variable was L/pig/hour. Water consumption was converted to mL to ensure it was on a similar scale to the other variables, avoiding potential bias in the model. Additionally, a descriptive analysis was performed by plotting the mean values of the variables ​​by hour of the day or by fattening day on time series graphs using the R package *ggplot2*. To characterise the daily cyclic pattern of water consumption, a harmonic decomposition approach was applied to model the cyclic daily pattern of water consumption, using a linear combination of sine and cosine terms corresponding to the four first harmonics of the 24 h cycle (24 h, 12 h, 8 h, and 6 h), following the approach described by Madsen et al^14,^ as described in their Materials and Methods section. Finally, differences in drinking patterns across hours of the day were assessed using a one-way ANOVA followed by Tukey HSD post-hoc tests to identify which hours differed significantly from each other.

### Model development

A time series model in the form of an ARMA process was used to describe and predict the water consumption pattern of growing pigs over the fattening period. This type of model assumes that the process at a given time t (in our case, at a given hour t) is autocorrelated with its own past (autoregressive (AR) terms) and that the random error process of the model may also be autocorrelated with its past as well (moving average (MA) terms), allowing it to model temporal dependencies of the process of interest^26^. In short, a (weakly) stationary time series process has a constant mean and variance, and its autocovariance function is independent of time and depends only on time lags^27^. Nevertheless, ARMA models cannot consider exogenous variables that may also affect water usage patterns, as they were originally designed to model stationary time series. In addition, in the presence of time series with trends and seasonal components, the property of stationarity would not be satisfied, and therefore ARMA models would not be appropriate. However, an extension of these models, the ARMAX models, allows ARMA models to incorporate exogenous variables as well as trends and seasonal components, and thus to model non-stationary time series with ARMA-type structures.

Accordingly, an ARMAX model structure was considered since allows the incorporation of exogenous variables that can potentially affect the temporal dynamics of the water consumption in fattening pigs. As the ARMAX model was used for both description and prediction purposes, the dataset was divided into two subsets. The first dataset was used to estimate the model structure—that is, to select the autoregressive orders for both the process and the random errors. It was also used to determine which covariates, among all candidates, were statistically significant for describing the evolution of water consumption in fattening pigs, as well as for prediction purposes. The second one was employed for predicting the short-term behaviour of water usage in fattening pigs. Overall, 24 time series (four batches per unit) were assigned to the first set of data used for model estimation (train set), representing 76.3% of all observations (58.896 h). The remaining six time series (one batch per unit) were reserved for prediction (test set), which consisted of 23.7% of total observations (18.642 h).

#### Auto-regressive moving average (ARMA) model

Literature describing ARMA models in detail are extensive^25,27–29^. Briefly, an ARMA model combines and autoregressive (AR) and a moving average (MA) component^27^. It assumes that the time series $\:\left\{{X}_{t}\right\}$ is stationary, meaning that: its expected value is constant over time $\:{\left(E\right(X}_{t})=\mu\:)$, its variance is finite $\:{\left(E\right(X}_{t}^{2})<\infty\:)$, and its autocovariance depends only on the temporal lag $\:h,$not on time $\:t$ $\:(Cov\left({X}_{t},{X}_{t+h}\right)=\gamma\:(h)$, where $\:\gamma\:\left(0\right)$ denotes the variance. Formally, ARMA models are defined as follows:

$$\begin{gathered} \:{X_t} = \mu \: + \:\phi {\:_1}{X_{t - 1}} + \ldots \: + \:\phi {\:_p}{X_{t - p}} + {\:_t} + \:\vartheta {\:_1}{\:_{t - 1}} + \ldots \: + \:\vartheta {\:_q}{\:_{t - q}} = \hfill \\ \: = \:\mu \: + \sum \: _{i = 1}^p\phi {\:_i}{X_{t - i}} + \sum \: _{i = 0}^q\vartheta {\:_i}{\:_{t - i}} \hfill \\ \end{gathered}$$

where $\:p>0$ and $\:q>0$ are the orders of the AR and MA part, respectively, and parameters $\:{\phi\:}_{1},\:\dots\:,\:{\phi\:}_{p}$ are associated with the AR part of the model while parameters $\:{\vartheta\:}_{1},\:\dots\:,\:{\vartheta\:}_{q}$ are related to the MA part of the model. In addition, parameter $\:\mu\:$ refers to the mean of $\:{X}_{t}$ for all $\:t$, $\:{\vartheta\:}_{0}=1$ and $\:{\epsilon\:}_{t}$ for all $\:t$ are independent and identically distributed random variables with $\:{E(\epsilon\:}_{t})=0$ and $\:{V(\epsilon\:}_{t})={\sigma\:}^{2}$, i.e., $\:{\epsilon\:}_{t}\sim\:WN(0,{\sigma\:}^{2})$ where $\:WN$ refers to white noise (30, 31). Note that if $\:q=0$, then the ARMA(p,0) model simplifies to an AR(p) model, while if $\:p=0$ the ARMA(0,q) model simplifies to a MA(q) model.

The parameters *p* and *q* of the ARMA model were determined to obtain the optimal AR ($\:\phi\:)$ and MA ($\:\theta\:)$ coefficients. Since for models with $\:p>0\:$and $\:q>0$ the sample autocorrelation function (ACF) and partial autocorrelation function (PACF) are difficult to recognize and are of far less value in order selection than in the special cases where $\:p=0$ or $\:q=0$, the order selection was performed through the minimization of the Akaike Information Criterion (AIC), Bayesian Information Criterion (BIC) and Corrected Akaike Information Criterion (AICc) statistics^28^. In brief, AIC and AICc balance model fit and complexity, penalizing excessive parameters to prevent overfitting, whereas BIC applies a stronger penalty for model complexity, favouring simpler models when sample size is large. Thus, lower values of these criteria indicate a better balance between goodness of fit and model simplicity.

In addition, parameter estimation for ARMA models, i.e., $\:{\Theta\:}=(\mu\:,\:{\phi\:}_{1},\:\dots\:,\:{\phi\:}_{p},\:{\vartheta\:}_{1},\dots\:{\vartheta\:}_{q},{\sigma\:}^{2})$, can be performed using either conditional least squares (CLS) or maximum likelihood (ML) methods. The CLS method estimates Θ by minimizing the sum of squared residuals—the differences between observed and model-predicted values. In contrast, the ML method estimates Θ by maximizing the model’s likelihood function, solved numerically. A common approach for parameter estimation in stationary ARMA models (e.g., the default procedure for the *arima* function in R) involves initially applying the CLS method. The resulting CLS estimates of Θ are then used as starting values for the numerical maximization of the model’s likelihood function. Lastly, to evaluate the statistical significance of the AR and MA parameters, we tested the null hypotheses $\:{\mathrm{H}}_{0}:{{\upphi\:}}_{\mathrm{i}}=0$ versus $\:{\mathrm{H}}_{1}:{{\upphi\:}}_{\mathrm{i}}\ne\:0$ for $\:\mathrm{i}=1,\:\cdots\:,\:\mathrm{p}\:$and $\:{\mathrm{H}}_{0}:{\:{\upvartheta\:}}_{\mathrm{i}}=0$ versus $\:{\mathrm{H}}_{1}:{{\upvartheta\:}}_{\mathrm{i}}\ne\:0$ for $\:\mathrm{i}=1,\:\cdots\:,\:\mathrm{q}$. In words, these hypotheses evaluate whether each parameter is statistically different from zero, meaning it provides meaningful explanatory power in the model. Wald significance tests were applied under the assumption that ML estimators are asymptotically normally distributed, being asymptotically not biased and efficient. The R packages *tidyverse*,* stats*,* timeSeries* and *forecast* were employed for data preprocessing, visualization, model fitting (*arima* function) and evaluation.

#### ARMAX: inclusion of external parameters

The notation ARMAX(*p*,* q*,*b*) refers to a model with *p* AR terms, *q* MA terms and *b* exogenous input terms. The last term is a linear combination of the last *b* terms of a known and external time series $\:{Y}_{t}$. It is given by:

$$\:{X}_{t}=\:\mu\:+\:{\epsilon\:}_{t}+{\sum\:}_{i=1}^{p}{\phi\:}_{i}{X}_{t-i}+{\sum\:}_{i=1}^{q}{\theta\:}_{i}{\epsilon\:}_{t-i}+\:{\sum\:}_{j=1}^{K}\:{\sum\:}_{i=1}^{b}{\eta\:}_{i,j}{Y}_{t-i,j}$$

where $\:{\eta\:}_{1,j},\:\dots\:\:,\:{\eta\:}_{b,j}$ are the coefficients of one of the *K* the exogenous inputs ($\:{Y}_{j}).$ For example, external variables such as temperature, ammonia and CO_2_ concentration, relative humidity and fattening day could be added to the model as external parameters, $\:{Y}_{t}$, considering the last *b* observations of each variable. As for the conventional ARMA, the conditional least squares and maximum likelihood methods were also used to estimate parameters, and AIC, AICc and BIC were also applied for model order selection.

#### Validation of the model

Several ARMAX model specifications were evaluated to determine the structure that best captured the dynamics of water consumption. Model selection was guided by RMSE (Root Mean Squared Error), which measures the average magnitude of prediction errors, as well as by AIC, AICc, and BIC. The final ARMAX configuration was chosen because it consistently achieved the lowest RMSE, AIC, AICc, and BIC values, demonstrating superior predictive accuracy and an appropriate balance between model fit and complexity. The validation of the model was carried out through a combination of statistical and graphical diagnostics. Coefficient significance was evaluated using t-tests, by computing the ratio of each estimated coefficient to its standard error. Residual analysis was performed to assess model adequacy, including tests for independence and normality. Specifically, the Ljung-Box test was applied to verify the absence of autocorrelation in the residuals. Normality was assessed both visually, through histograms and density plots, and statistically, using the Shapiro-Wilk test. A plot of residuals *versus* fitted values was also generated to evaluate homoscedasticity and identify potential model misspecification. These procedures ensured that the model accurately reflected the underlying data structure and satisfied the assumptions necessary for robust and reliable inference.

## Results

### Drinking pattern in fattening pigs

**Fig. 1:**
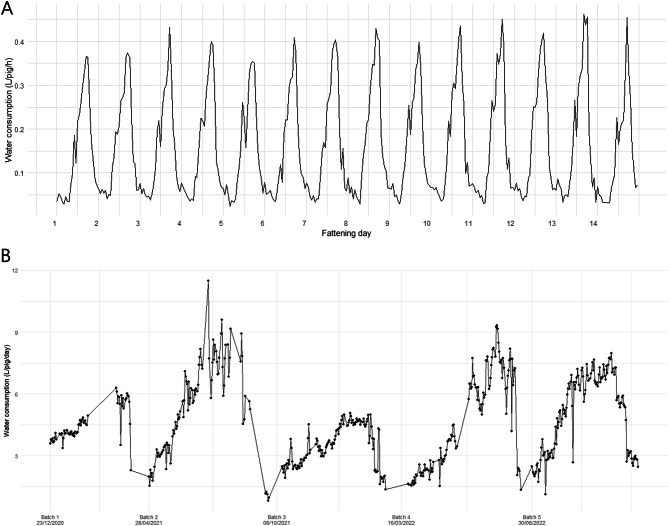
Water consumption in litres per pig and hour (L/pig/h) of a single batch (Batch 1) recorded from 23/12/2020 to 05/01/2021 during the first two weeks of fattening (**A**), and daily water consumption in litres per pig per day (L/pig/day) of five consecutive batches from one barn (**B**).

In this study, daily drinking water for pigs ranged from an average of 2.35 ± 0.54 L/pig/day to 8.05 ± 2.17 L/pig/day through the fattening period. A stable daily oscillation, i.e., circadian rhythm, was observed in the water consumption, with a diurnal pattern where the level of water consumed was minimal during the night (Fig. 1A). Within a batch, the water consumption per pig showed an increasing trend as pigs grew, followed by a sharp decrease at the end of the fattening period until a new batch of pigs entered the farm (Fig. 1B). Consequently, the total water consumption rate increased throughout the fattening period, since pigs drink more as they grow.

**Fig. 2:**
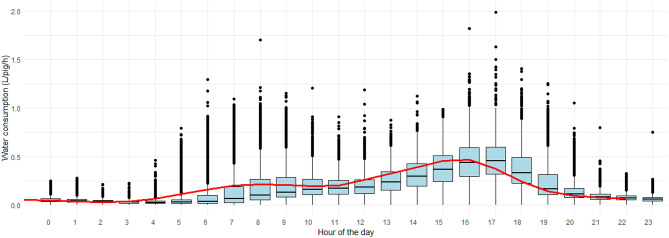
Boxplot and mean (red line) of the hourly water consumption (L/pig/h) in one day considering data of 30 time series (i.e., six barns and five batches of fattening pigs).

**Fig. 3:**
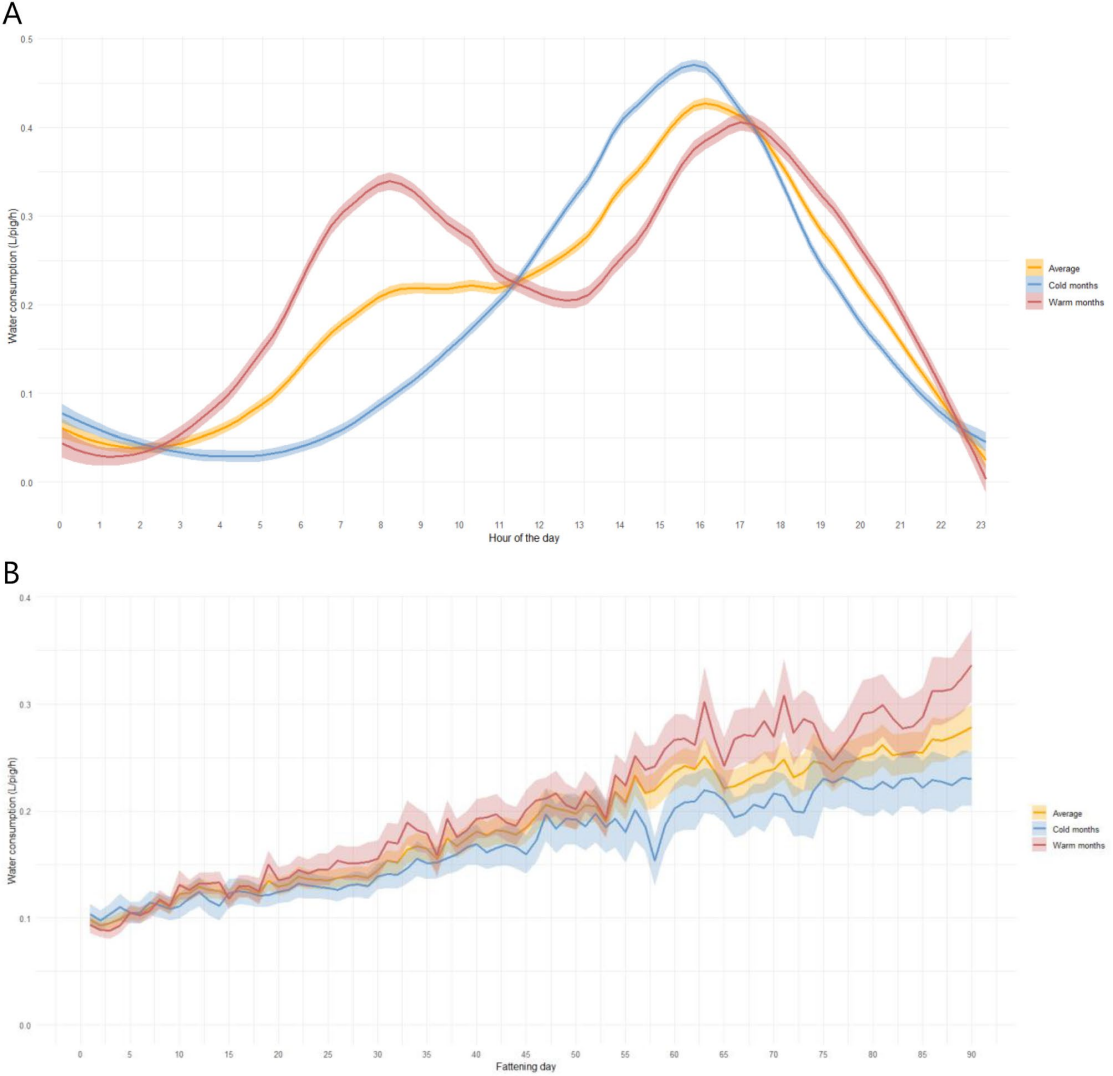
Mean water consumption (L/pig/h) per hour of the day (**A**) and per fattening day over a period of 90 days (**B**) in hot (May–October) and cold months (November–April). The drinking pattern during cold months is shown in blue, while during hot months it is shown in red. The orange line represents the overall average water consumption, based on data from 30 time series (i.e., six barns and five batches of fattening pigs). Shaded areas around the lines indicate 95% confidence intervals for the mean estimates.

Considering all data and within a given day, fattening pigs began drinking water around 5 to 6 a.m., with water consumption increasing and reaching higher values during the morning hours, particularly between 8 a.m. and 12 p.m. (e.g., 0.22 ± 0.20 L/pig/h at 9 a.m.), and again during the late afternoon between 4 and 5 p.m. (e.g., 0.47 ± 0.23 L/pig/h at 5 p.m.). Thereafter, consumption gradually declined for the rest of the day (Fig. 2). A two-way ANOVA confirmed that the hour of the day significantly influenced the drinking pattern ($\:F(23,\:77184)\:=\:2780$, $\:p\:<\:2e-16$). Tukey HSD post-hoc tests showed that water consumption did not differ significantly within the morning (8–12 a.m.) or late-afternoon (4–5 p.m.) periods, whereas both periods differed significantly from most other hours of the day. Besides, when data collected during hot months (May – October) were plotted against data collected during the cold months (November – April) of the year, two patterns of water consumption were discerned (Fig. 3A). See Table S3 with farm’s monthly temperature data in Supplementary Material. During hot months, the two peaks in consumption previously noted remained, being the first one at 8 to 9 a.m. more pronounced, whereas in cold months, the first peak was nearly absent. Furthermore, temperature outside the farm appeared to influence not only the circadian rhythm but also the overall trend in water consumption, which was steeper in hot temperatures (Fig. 3B).

### Trend and seasonality

**Fig. 4:**
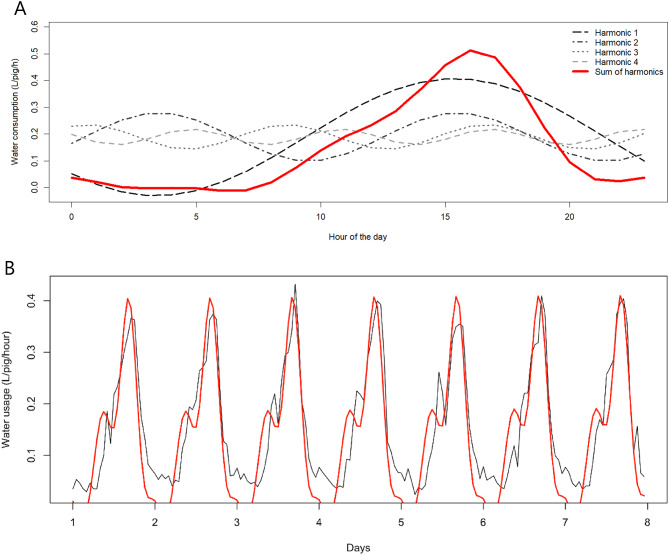
The diurnal drinking pattern (black line) is shown together with the four harmonic waves; 24 h (H1), 12 h (H2), 8 h (H3) and 6 h (H4). The sum of the four harmonic components forms the cyclic pattern, indicated by the solid line in black for one day (**A**) or red for one week (**B**) of fattening.

The harmonic decomposition revealed a cyclic pattern in the daily water consumption of fattening pigs. The first harmonic captured the main 24-h rhythm, while the higher-order harmonics accounted for the additional fluctuations observed throughout the day, resulting in a smooth and well-defined representation of the drinking cycle (Figs. 4A − 4B).

Moreover, as pigs consume greater amounts of water as they grow, the fattening day (i.e., days elapsed since the start of the fattening period) was included as a key explanatory variable to capture and model this progressive trend. This approach allowed for a quantitative representation of the relationship between growth and drinking behaviour over time, enabling a more accurate understanding of how water consumption scales with the pigs’ maturation and overall body mass increase.

### ARMA model

Based on the lowest AIC value, an ARMA (24, 24) was selected. The t-tests revealed that several coefficients were not statistically significant ($\:p>0.05$) and were nearly zero; these were consequently fixed to zero. The final ARMA model is summarized in Table 1, which reports the non-zero AR and MA coefficients along with their associated standard errors. The table provides a clear overview of the model’s dynamic components, capturing the temporal structure of the time series.

**Table 1:** Estimated coefficients and standard errors for the non-zero AR and MA terms of the model. Only the parameters retained in the final model specification are included.

Term	Coefficient	Standard Error
$\:{\phi\:}_{1}$	1.1045	0.1008
$\:{\phi\:}_{2}$	−0.3326	0.0454
$\:{\phi\:}_{22}$	−0.2183	0.0370
$\:{\phi\:}_{23}$	0.8993	0.0666
$\:{\phi\:}_{24}$	−0.4818	0.0744
$\:{\vartheta\:}_{1}$	−0.6963	0.1033
$\:{\vartheta\:}_{2}$	0.1583	0.0223
$\:{\vartheta\:}_{22}$	0.2733	0.0411
$\:{\vartheta\:}_{23}$	−0.7566	0.0554
$\:{\vartheta\:}_{24}$	0.2893	0.0625

### ARMAX model

In addition to the AR and MA components previously discussed, external variables were incorporated into the model as exogenous inputs, as their coefficients were statistically significant based on t-tests ($\:p\:<\:0.05$) and contributed to improving predictive accuracy. The external variables included environmental temperature outside the farm, ammonia concentration, and fattening day, which, together with the selected ARMA model orders, resulted in the model with the lowest AIC, AICc and BIC values (AIC = 1494.97, AICc = 1495.58, BIC = 1629.25). Table 2 summarizes the estimated coefficients of these external parameters, and the harmonics included in the ARMAX model, along with their associated standard errors. The sign of each coefficient reflects its adjusted effect on water consumption within the multivariable ARMAX model.

**Table 2:** Estimated coefficients and standard errors for the external regressors and harmonic terms of the model. Only the parameters retained in the final model specification are included.

Term	Description	Coefficient	Standard Error
S1 ($\:{\eta\:}_{\mathrm{1,1}}$)	Sine (period 24 h)	−0.7513	0.0660
S2 ($\:{\eta\:}_{\mathrm{2,1}}$)	Sine (period 12 h)	−0.4167	0.0417
S3 ($\:{\eta\:}_{\mathrm{3,1}}$)	Sine (period 8 h)	0.2275	0.0263
S4 ($\:{\eta\:}_{\mathrm{4,1}}$)	Sine (period 6 h)	0.1351	0.0161
C1 ($\:{\eta\:}_{\mathrm{5,1}}$)	Cosine (period 24 h)	−0.7394	0.0523
C2 ($\:{\eta\:}_{\mathrm{6,1}}$)	Cosine (period 12 h)	−0.1976	0.0395
C3 ($\:{\eta\:}_{\mathrm{7,1}}$)	Cosine (period 8 h)	0.1960	0.0265
C4 ($\:{\eta\:}_{\mathrm{8,1}}$)	Cosine (period 6 h)	0.0482	0.0158
T ($\:{\eta\:}_{\mathrm{9,1}}$)	Outside temperature	0.0198	0.0055
NH_3_ ($\:{\eta\:}_{\mathrm{10,1}}$)	Ammonia concentration	−0.0142	0.0058
N ($\:{\eta\:}_{\mathrm{11,1}}$)	Fattening day	−0.1196	0.0325

**Fig. 5:**
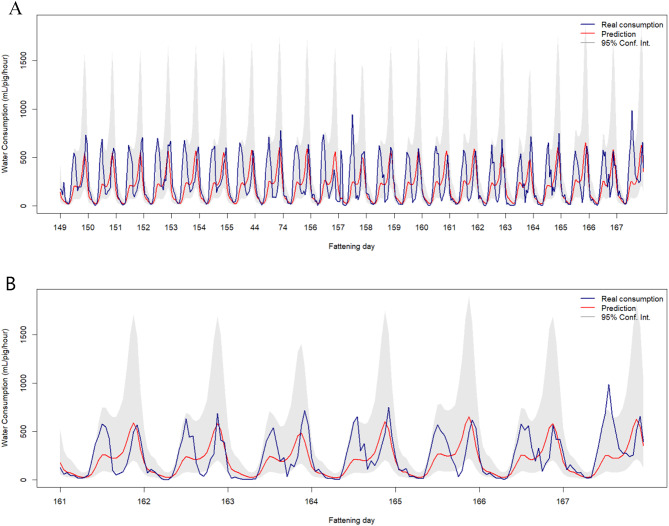
Real water consumption (blue) against the predicted values (red) of one batch for a three-week period (**A**) and one week period (**B**) during the fattening cycle. Shaded grey areas represent the 95% confidence intervals around the predicted values.

Using the *forecast* function in R, predictions of water consumption were generated using the ARMAX(24,24) model for a batch over the fattening period. Point forecasts were accompanied by their corresponding 95% prediction intervals. Figures 5A and 5B displays the predicted water intake values (in red) alongside the actual recorded consumption (in blue), clearly illustrating the model’s performance during this validation window. Notably, most of the observed data fell within the 95% prediction intervals, indicating that the model was able to reliably capture the variability in water consumption and produce accurate forecasts.

### Statistical model checking

**Fig. 6:**
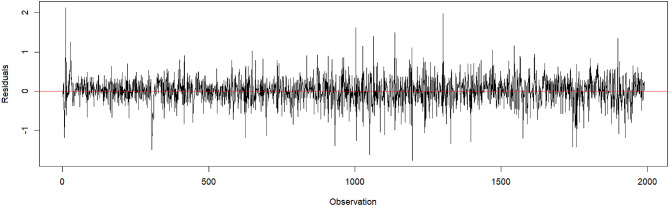
Plot of the standardized forecast errors of the predictions of one batch. The plot does not indicate any changes over time.

The standardized forecast errors clustered around zero, indicating that the model did not exhibit evident systematic bias and provided accurate predictions across the evaluated batches (Fig. 6). For additional validation of the selected ARMAX model, see Figures S1 and S2 in the Supplementary Material. The histogram of the residuals (Figure S1) and the QQ plot (Figure S2) suggest that the residuals are approximately normally distributed. However, the Shapiro-Wilk normality test yielded a W statistic of 0.97 with $\:p\:<\:0.01$ indicating a slight deviation from normality. Additionally, the Ljung-Box test for autocorrelation showed an X-squared value of 17.53 with 24 degrees of freedom and $\:p\:=\:0.825$, suggesting no significant autocorrelation in the residuals up to lag 24. Overall, the final model showed an RMSE of 88.87, reflecting the average prediction error in the same units as the observed data.

## Discussion

Livestock production has increasingly adapted to meet social demands for sustainability, animal welfare, and reduced environmental impact. This shift has highlighted the importance of continuous monitoring throughout animal rearing. For instance, modelling and daily charting of drinking water usage can serve as a predictor of the onset of swine health and welfare challenges^11,12^. In the same line, the present study provided a detailed analysis of water consumption behaviour in fattening pigs based on an ARMAX model structure, including the average daily drinking pattern, patterns related to trends and seasonal components, as well as potential structures of autocorrelation of the water consumption process. The ARMAX model was also used to forecast water intake on an hourly basis. By incorporating external factors such as temperature, ammonia concentration, and days of fattening, the model improved predictive performance and captured part of the temporal structure of drinking behaviour, particularly its trend and daily variation. Monitoring such patterns is an essential first step for utilizing drinking behaviour as a reliable early warning system of health and welfare challenges affecting pigs reared under commercial conditions.

Published studies on the daily water use patterns of pigs have varied widely in cohort size and study duration. While a large majority of them used water flowmeters to describe water use volumetrically^11,14^, some others relied on video recordings to measure time spent drinking^31,32 ^or used a radio frequency identification drinking system to count the number of water intake visits^33^. More recently, machine vision and deep learning approaches have been applied to automatically identify individual pigs and detect drinking behaviours in group-housed systems^34–37^. In contrast, the present work, which spanned three years and included hourly water usage data at the barn level across 30 time series, comprising more than 77.538 observations from mostly healthy pigs, provides an unprecedented scale and temporal granularity using data that are straightforward to collect, computationally inexpensive to process, and readily available under commercial production conditions.

Water intake is influenced by both intrinsic factors, such as body weight and feeding schedules, and extrinsic factors, including temperature, air quality, and location of the drinkers^16,32,38^. Results from this study revealed a progressive increase in water consumption throughout the fattening period associated with pigs’ growth. Water use by pigs is indeed a function of their body weight. This has been measured with various combinations of drinker types, heights, and water flow rates, and averages between a little less than 6 L/pig/day to a bit more than 8 L/pig/day for grow-finishing pigs, which is consistent with our results^15,39–41^. Furthermore, our data demonstrated that water use by pigs is influenced by the time of day. Pigs primarily consumed water during daylight hours, with consumption increasing during the morning (8 a.m. – 12 p.m.) and again during the late afternoon (16–17 p.m.), reflecting a circadian pattern. This finding is consistent with previous studies reporting pronounced circadian and diurnal rhythms in pig behaviour under commercial and experimental conditions, including daily patterns in feeding activity^11,31,42,43^ as well as other physiological and behavioural processes^44,45^. Such rhythms have been shown to be shaped by endogenous circadian regulation as well as by external factors such as feeding schedules and light–dark cycles, supporting the interpretation that the temporal structure observed in water use reflects broader daily activity rhythms in pigs.

The inclusion of feed intake as a predictor of water consumption in growing pigs is indeed supported by a well-documented physiological relationship between both variables. Typical water-to-feed ratios range from 1.5:1 to 2.8:1 in pigs fed dry feed *ad libitum*^46^, indicating that increased feed intake is generally accompanied by higher water consumption. These ratios also decline as pigs grow, reinforcing the dynamic association between both variables. However, despite its biological relevance, feed intake was excluded from the ARMAX model due to unreliable hourly data collected. While laser sensors are excellent for monitoring general trends and silo levels, their temporal resolution, measurement noise, and indirect estimation of mass make them unsuitable for precise hourly feed consumption tracking without additional corrections and data processing. For instance, silos are refilled periodically, and unless sensor data are corrected for these events, they can distort consumption curves. Also, refills may happen during the hour and obscure actual consumption rates. For more precise monitoring of feed intake, alternative methods such as integrating load cells with feeding systems or using smart feeders that record individual animal consumption may be more appropriate.

During warm temperature conditions, pigs consume more water to compensate for their limited ability to dissipate heat through evaporation and to prevent heat stress, which negatively affects feed intake and overall performance^47^. Conversely, under colder conditions, pigs increase feed consumption and decrease water intake to support thermogenesis and maintain body warmth^47^. Our results suggest that seasonal differences – specifically between warm and cold periods – are associated with changes in the 24-hour water usage pattern in grow-finishing pigs. As reported by Brumm (2006), during warm season, morning and afternoon consumption peaks are pronounced, while in colder months, the morning peak is nearly absent and appears earlier^42^. These seasonal variations emphasize the need to consider external environmental variables when interpreting water consumption data. This is particularly relevant for practical applications, as it minimizes the risk of confounding health-related deviations with environmental influences. For example, deviations in water consumption during heat stress, as noted by Matthews et al. (2016), could otherwise be misinterpreted as health concerns without contextual environmental data. Although outside temperature differs from the barn’s internal climate, outdoor temperature was used because it showed a stronger association with water-consumption patterns. Given the high correlation between indoor and outdoor temperatures, including both would have introduced redundancy without improving predictive accuracy; therefore, outdoor temperature was retained as the most informative predictor.

In addition to temperature, other environmental factors—particularly air quality—may influence water consumption in fattening pigs^16,24^. Under the conditions of the present study, ammonia concentration was an explanatory variable for water intake. Previous research has shown that ammonia levels exceeding 100 ppm can impair feed intake and reduce growth performance^48,49^. Moreover, chronic exposure to lower concentrations has been associated with respiratory disorders and behavioural alterations, including increased tail biting and changes in activity behaviour^50–52^. It should be noted, however, that in this study, ammonia concentrations were probably confounded with other environmental variables, such as ambient temperature and CO₂ levels. For instance, ammonia concentrations were substantially lower during the summer months, likely due to enhanced natural ventilation from open barn windows. Thus, the association between ammonia and water consumption may partly reflect overall air-quality conditions rather than a purely isolated effect. Nevertheless, ammonia was included in the final model because its coefficient was statistically significant and it improved predictive performance. Other air-quality parameters, although available in the database, were not included in the model. In the case of CO₂, the sensor registered frequent failures and long periods without valid readings, preventing its reliable use as a predictor. Relative humidity was excluded due to redundancy and lack of statistical significance.

ARMAX models are relatively easy to interpret and can be applied both to describe the temporal dynamics of a process and to generate reliable short-term forecasts. They are also efficiently implemented in various statistical software packages, such as R. In this study, an ARMAX(24,24) model was developed to describe the temporal evolution of water consumption in fattening pigs, capturing both trend and cyclical components, as well as the association with external variables. Specifically, the model integrated AR and MA terms with exogenous covariates, allowing to capture both short-term fluctuations and broader behavioural patterns. In addition, fattening day and harmonic components were effective in modelling long-term trends and daily seasonality. Once fitted, the model was also used as a predictive tool, successfully forecasting hourly water usage over a one-week period. Most observed values fell within the 95% confidence interval of the forecast, and the standardized residuals were symmetrically distributed around zero, indicating an overall satisfactory model performance across batches under the studied conditions. However, the model tended to underestimate the first peak in observed water consumption, which highlights a limitation in its ability to fully capture short-term increases in drinking behaviour. This discrepancy may be partly explained by the imbalance in the training data, which included a higher number of batches raised during winter compared to summer. On the other hand, although included in the model, temperature effects may not fully account for seasonal or management-related influences on early drinking patterns. Despite this limitation, the predictive approach remains valuable for the early detection of anomalies in water consumption that may indicate health or welfare issues, thereby supporting more proactive and sustainable management strategies in commercial swine production systems.

Defining the water requirement of the pig is particularly challenging because intake can be affected by several metabolic, physiological and behavioural factors, and *ad libitum* intake is not always a reflection of need^47^. Indeed, differences in the water use pattern within each day of pig cohorts across buildings and seasons have already been reported^43^. The study’s scope is limited to data from a single farm under specific environmental conditions, which restricts the generalizability of the model to other settings. In particular, the results should be interpreted within the context of the management practices, housing design, climate and genetic lines present in the studied farm, as these factors can substantially influence water consumption patterns. Future research incorporating multiple farms and diverse environmental context would therefore be essential to validate the model and asses its robustness across production systems. Additionally, exploring the integration of other advanced algorithms could further enhance the model’s predictive accuracy and adaptability. For instance, coupling dynamic or temporal statistical models with machine learning algorithms could overcome some of the limitations of our ARMAX-based approach by capturing non-linear dependencies and complex interactions that ARMAX may not fully model^32,39^. Moreover, alternative time series models such as ARIMA could also be tested to compare performance^53^. This integration would allow for a more adaptive framework that leverages both past water consumption observations and external parameters to enhance the early detection of subtle patterns preceding health or welfare challenges.

Our study shows that pig drinking behaviour is influenced by both intrinsic and environmental factors and follows a consistent circadian pattern, with daily peaks in water consumption across all analysed batches. The large, high-resolution dataset collected over three years supports the use of predictive models such as ARMAX and highlights the importance of including environmental variables to improve their accuracy. By capturing deviations from expected patterns, this model can potentially support the development of early warning systems that can help to identify farm management challenges such as heat stress or illness before clinical signs become apparent.

## Conclusions

The present research contributes to a better understanding of the water consumption behaviour of growing pigs across the fattening period by characterizing both the increasing trend over time and the daily consumption pattern. We developed an ARMAX model including seasonal components, historic water intake observations, and external variables that was proven to be well suited for modelling purposes under the specific conditions of the studied farm. Given the single-site nature of the dataset, further validation across farm with different conditions would be essential to assess the model’s broader applicability. Ultimately, the monitoring of water consumption patterns is a prerequisite for utilizing drinking behaviour for an early detection of health and welfare issues in growing pigs.

## Supplementary Information

Below is the link to the electronic supplementary material.

Supplementary Material 1

## Data Availability

The data that support the findings of this study are available from Grupo Vall Companys but restrictions apply to the availability of these data, which were used under license for the current study, and so are not publicly available. Data are however available from the authors upon reasonable request and with permission of Grupo Vall Companys. Requests for data should be directed to Dr. Beatriz Garcia-Morante at [beatriz.garciam@irta.cat](mailto: beatriz.garciam@irta.cat).The code used to develop and run the ARMAX model in this study is publicly available in a GitHub repository at: [https://github.com/mariamarrocorios/armax.git](https:/github.com/mariamarrocorios/armax.git).

## Abbreviations

ARMA: Autoregressive moving average
ARMAX: Autoregressive moving average with exogenous inputs
AR: Autoregressive
MA: Moving average
PLF: Precision livestock farming
DLM: Dynamic linear model
ACF: Autocorrelation function
PACF: Partial autocorrelation function
AIC: Akaike information criterion
BIC: Bayesian information criterion
AICc: Corrected akaike information criterion
CLS: Conditional least squares
ML: Maximum likelihood
RMSE: Root mean square error
