# Supplementary material for: Modeling and predicting water consumption in fattening pigs using autoregressive moving average with external parameters
Source: Sci Rep. 2026 Feb 20;16:10027. doi: 10.1038/s41598-026-40343-7 (PMC13022239; doi:10.1038/s41598-026-40343-7)
Supplement: Supplementary file 1 — Supplementary Material 1 [file 41598_2026_40343_MOESM1_ESM.docx]

**Supplementary Material**

**Table S1.** Summary of batch size and recording periods for the five production batches included in the study.

| **Batch** | **Number of pigs** | **Average Pigs per Barn (Initial)** | **Start Date** | **End Date** |
| --- | --- | --- | --- | --- |
| Batch 1 | 4296 | 716 | 23/12/2020 | 13/04/2020 |
| Batch 2 | 4212 | 702 | 28/04/2021 | 28/09/2021 |
| Batch 3 | 4002 | 667 | 08/10/2021 | 27/02/2022 |
| Batch 4 | 4080 | 680 | 16/03/2022 | 17/08/2022 |
| Batch 5 | 4368 | 728 | 30/08/2022 | 16/01/2023 |

**Table S2.** Percentage of outliers (time points where water intake was higher than 10 L/pig/hour) and missing values (observations where water usage did not match a number of pigs or *vice versa*) per batch and building. These observations were not included in the model.

| **Building** | **Batch** | **Outliers (%)** | **Missing values (%)** |
| --- | --- | --- | --- |
| A | 1 | 1.11 | 56.29 |
|  | 2 | 21.14 | 5.38 |
|  | 3 | 4.03 | 18.95 |
|  | 4 | 8.66 | 14.77 |
|  | 5 | 3.68 | 8.72 |
| B | 1 | 1.02 | 50.99 |
|  | 2 | 19.13 | 11.54 |
|  | 3 | 5.65 | 18.95 |
|  | 4 | 8.15 | 9.40 |
|  | 5 | 3.60 | 6.71 |
| C | 1 | 1.09 | 54.97 |
|  | 2 | 19.44 | 16.92 |
|  | 3 | 3.91 | 16.34 |
|  | 4 | 8.53 | 13.42 |
|  | 5 | 3.70 | 9.40 |
| D | 1 | 1.02 | 50.99 |
|  | 2 | 21.55 | 10.77 |
|  | 3 | 3.91 | 16.34 |
|  | 4 | 8.59 | 14.09 |
|  | 5 | 3.57 | 6.04 |
| E | 1 | 1.03 | 51.66 |
|  | 2 | 19.64 | 13.85 |
|  | 3 | 3.73 | 12.42 |
|  | 4 | 7.46 | 10.07 |
|  | 5 | 3.82 | 10.88 |
| F | 1 | 1.02 | 50.99 |
|  | 2 | 20.54 | 13.85 |
|  | 3 | 3.73 | 12.42 |
|  | 4 | 8.03 | 8.05 |
|  | 5 | 3.82 | 10.88 |

**Table S3.** Monthly mean and standard deviation of outdoor and indoor temperatures recorded across the study period.

| **Month** | **Outdoor Temp (mean, ^o^C)** | **Outdoor Temp (SD)** | **Indoor Temp (mean, ^o^C)** | **Indoor Temp (SD)** |
| --- | --- | --- | --- | --- |
| January | 5.05 | 4.51 | 21.7 | 0.819 |
| February | 9.84 | 4.27 | 21.3 | 1.16 |
| March | 11.2 | 5.07 | 22.5 | 1.62 |
| April | 12.9 | 5.79 | 23.4 | 1.71 |
| May | 19.8 | 5.98 | 25.8 | 2.13 |
| June | 24.7 | 5.74 | 27.3 | 2.73 |
| July | 26.9 | 5.49 | 28.2 | 2.94 |
| August | 26.4 | 5.76 | 27.9 | 2.93 |
| September | 22.1 | 4.98 | 26.8 | 2.19 |
| October | 18.1 | 4.58 | 25.7 | 1.31 |
| November | 10.7 | 4.73 | 23.5 | 0.932 |
| December | 7.2 | 3.77 | 22.5 | 0.898 |


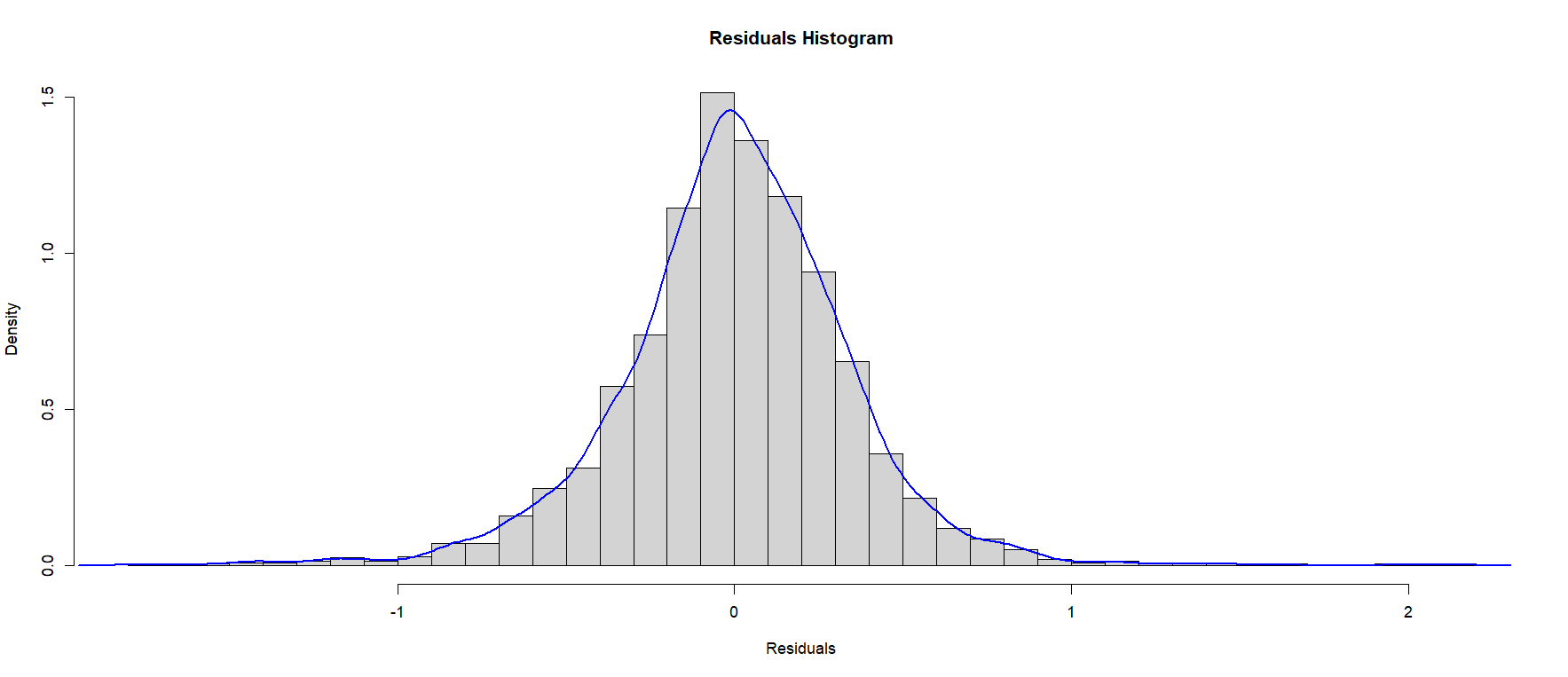


**Figure S1.** Histogram of the residuals from the ARMAX model. The plot shows the distribution of the residuals, which appear approximately normal, supporting the assumption of normality for the model's residuals.


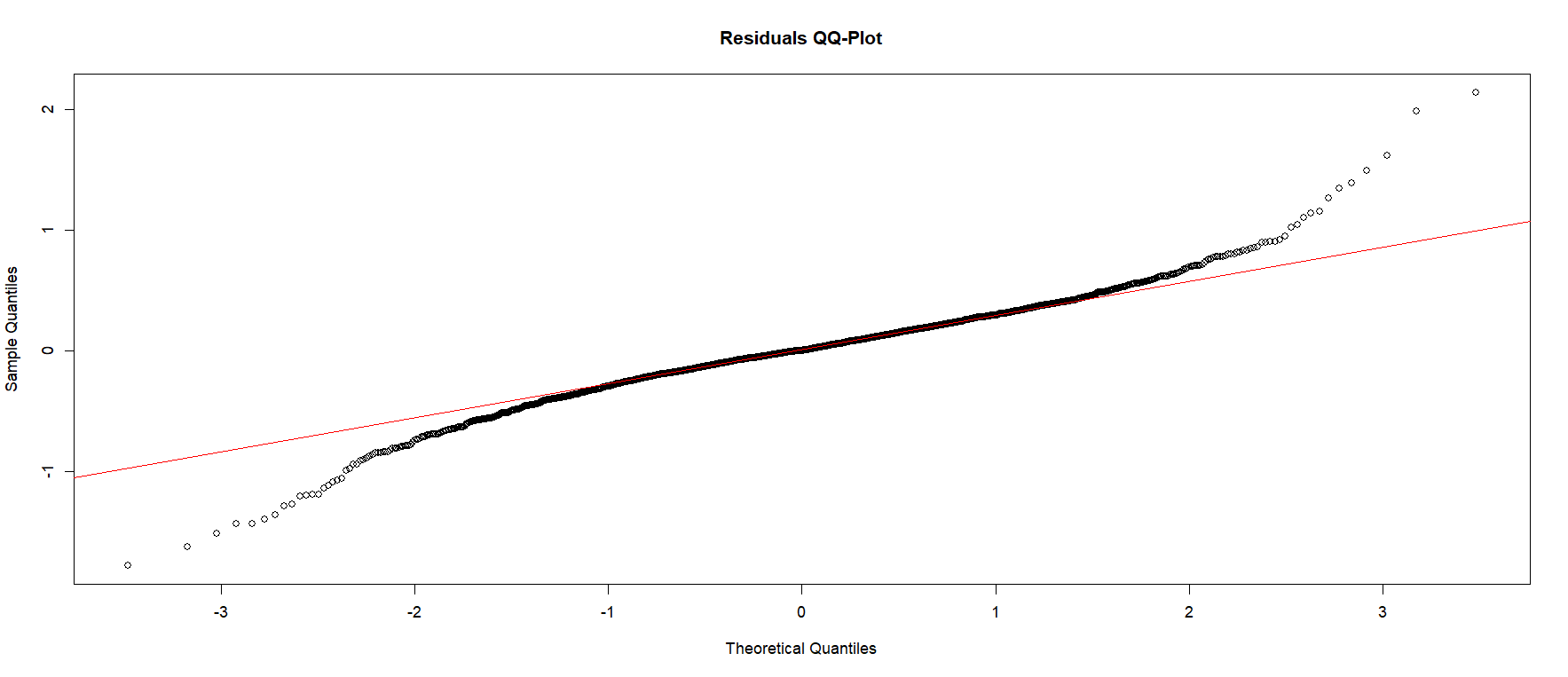


**Figure S2.** QQ plot of the residuals from the ARMAX model. The plot indicates that the residuals roughly follow a straight line, suggesting that they are approximately normally distributed, although the Shapiro-Wilk test suggests a slight departure from normality (p-value < 0.01).
